# Supplementary material for: Modeling glioblastoma heterogeneity as a dynamic network of cell states
Source: Mol Syst Biol. 2021 Sep 16;17(9):e10105. doi: 10.15252/msb.202010105 (PMC8444284; doi:10.15252/msb.202010105)
Supplement: Supplementary file 5 — Source Data for Figure 3 [file MSB-17-e10105-s001.zip › Figure3A_sourcedata/GSEA_3065/hallmarks_state6.GseaPreranked.1623416308702/HALLMARK_MYC_TARGETS_V1.html]

Details for gene set HALLMARK\_MYC\_TARGETS\_V1[GSEA]

|  || Dataset | state6 |
| Phenotype | NoPhenotypeAvailable |
| Upregulated in class | na\_pos |
| GeneSet | HALLMARK\_MYC\_TARGETS\_V1 |
| Enrichment Score (ES) | 0.28901425 |
| Normalized Enrichment Score (NES) | 1.3539226 |
| Nominal p-value | 0.012269938 |
| FDR q-value | 0.18272914 |
| FWER p-Value | 0.608 |
Table: GSEA Results Summary

  

Fig 1: Enrichment plot: HALLMARK\_MYC\_TARGETS\_V1      
 Profile of the Running ES Score & Positions of GeneSet Members on the Rank Ordered List

  

| PROBE | GENE SYMBOL | GENE\_TITLE | RANK IN GENE LIST | RANK METRIC SCORE | RUNNING ES | CORE ENRICHMENT || 1 | RPLP0 |  |  | 10 | 0.312 | 0.0207 | Yes |
| 2 | PABPC1 |  |  | 15 | 0.292 | 0.0407 | Yes |
| 3 | RPL22 |  |  | 26 | 0.274 | 0.0587 | Yes |
| 4 | RPL18 |  |  | 36 | 0.256 | 0.0756 | Yes |
| 5 | RPL6 |  |  | 38 | 0.254 | 0.0933 | Yes |
| 6 | RPL34 |  |  | 41 | 0.251 | 0.1106 | Yes |
| 7 | RACK1 |  |  | 57 | 0.240 | 0.1257 | Yes |
| 8 | RPS3 |  |  | 59 | 0.238 | 0.1422 | Yes |
| 9 | RPS5 |  |  | 92 | 0.218 | 0.1538 | Yes |
| 10 | SET |  |  | 100 | 0.213 | 0.1679 | Yes |
| 11 | HNRNPA1 |  |  | 108 | 0.209 | 0.1817 | Yes |
| 12 | TXNL4A |  |  | 154 | 0.190 | 0.1897 | Yes |
| 13 | RPS2 |  |  | 156 | 0.189 | 0.2029 | Yes |
| 14 | RPL14 |  |  | 192 | 0.178 | 0.2113 | Yes |
| 15 | ODC1 |  |  | 222 | 0.169 | 0.2197 | Yes |
| 16 | HDGF |  |  | 247 | 0.162 | 0.2283 | Yes |
| 17 | HNRNPC |  |  | 331 | 0.147 | 0.2289 | Yes |
| 18 | EIF2S2 |  |  | 347 | 0.144 | 0.2373 | Yes |
| 19 | LSM2 |  |  | 356 | 0.142 | 0.2463 | Yes |
| 20 | RFC4 |  |  | 397 | 0.136 | 0.2512 | Yes |
| 21 | GSPT1 |  |  | 455 | 0.127 | 0.2535 | Yes |
| 22 | YWHAQ |  |  | 478 | 0.125 | 0.2597 | Yes |
| 23 | PWP1 |  |  | 544 | 0.119 | 0.2605 | Yes |
| 24 | CLNS1A |  |  | 545 | 0.118 | 0.2688 | Yes |
| 25 | NHP2 |  |  | 598 | 0.114 | 0.2707 | Yes |
| 26 | STARD7 |  |  | 635 | 0.111 | 0.2743 | Yes |
| 27 | RPS6 |  |  | 647 | 0.110 | 0.2808 | Yes |
| 28 | CCT2 |  |  | 792 | 0.099 | 0.2710 | Yes |
| 29 | SNRPD2 |  |  | 805 | 0.098 | 0.2764 | Yes |
| 30 | PHB2 |  |  | 828 | 0.096 | 0.2806 | Yes |
| 31 | CBX3 |  |  | 966 | 0.087 | 0.2708 | Yes |
| 32 | CNBP |  |  | 997 | 0.086 | 0.2733 | Yes |
| 33 | IMPDH2 |  |  | 1043 | 0.083 | 0.2739 | Yes |
| 34 | AP3S1 |  |  | 1075 | 0.082 | 0.2760 | Yes |
| 35 | FAM120A |  |  | 1110 | 0.080 | 0.2777 | Yes |
| 36 | GLO1 |  |  | 1161 | 0.077 | 0.2773 | Yes |
| 37 | NOLC1 |  |  | 1181 | 0.076 | 0.2804 | Yes |
| 38 | CUL1 |  |  | 1195 | 0.075 | 0.2842 | Yes |
| 39 | TARDBP |  |  | 1200 | 0.075 | 0.2890 | Yes |
| 40 | LSM7 |  |  | 1254 | 0.073 | 0.2880 | No |
| 41 | PHB |  |  | 1374 | 0.068 | 0.2789 | No |
| 42 | CCT7 |  |  | 1407 | 0.067 | 0.2799 | No |
| 43 | CDK4 |  |  | 1490 | 0.064 | 0.2749 | No |
| 44 | DEK |  |  | 1535 | 0.063 | 0.2742 | No |
| 45 | POLD2 |  |  | 1589 | 0.061 | 0.2723 | No |
| 46 | EEF1B2 |  |  | 1665 | 0.058 | 0.2676 | No |
| 47 | EIF4H |  |  | 1671 | 0.058 | 0.2711 | No |
| 48 | USP1 |  |  | 1712 | 0.056 | 0.2704 | No |
| 49 | HSPE1 |  |  | 1803 | 0.054 | 0.2637 | No |
| 50 | SLC25A3 |  |  | 1835 | 0.052 | 0.2637 | No |
| 51 | EIF4G2 |  |  | 1849 | 0.052 | 0.2659 | No |
| 52 | RPS10 |  |  | 1934 | 0.050 | 0.2596 | No |
| 53 | SNRPG |  |  | 1951 | 0.049 | 0.2612 | No |
| 54 | CCT4 |  |  | 1969 | 0.049 | 0.2626 | No |
| 55 | PRPS2 |  |  | 2001 | 0.048 | 0.2623 | No |
| 56 | ERH |  |  | 2003 | 0.048 | 0.2656 | No |
| 57 | PSMA7 |  |  | 2029 | 0.047 | 0.2660 | No |
| 58 | MYC |  |  | 2081 | 0.045 | 0.2632 | No |
| 59 | NAP1L1 |  |  | 2171 | 0.043 | 0.2559 | No |
| 60 | NCBP1 |  |  | 2289 | 0.039 | 0.2450 | No |
| 61 | CCT3 |  |  | 2419 | 0.036 | 0.2325 | No |
| 62 | MCM6 |  |  | 2484 | 0.034 | 0.2274 | No |
| 63 | PABPC4 |  |  | 2558 | 0.032 | 0.2211 | No |
| 64 | PPM1G |  |  | 2588 | 0.031 | 0.2199 | No |
| 65 | PCBP1 |  |  | 2601 | 0.031 | 0.2207 | No |
| 66 | COPS5 |  |  | 2629 | 0.030 | 0.2197 | No |
| 67 | SF3B3 |  |  | 2648 | 0.030 | 0.2197 | No |
| 68 | UBE2E1 |  |  | 2722 | 0.028 | 0.2132 | No |
| 69 | DDX18 |  |  | 2755 | 0.028 | 0.2114 | No |
| 70 | GOT2 |  |  | 2783 | 0.027 | 0.2102 | No |
| 71 | PSMD14 |  |  | 2855 | 0.025 | 0.2037 | No |
| 72 | MRPL23 |  |  | 2860 | 0.025 | 0.2050 | No |
| 73 | EIF3J |  |  | 2953 | 0.023 | 0.1959 | No |
| 74 | XPOT |  |  | 2982 | 0.022 | 0.1942 | No |
| 75 | SRSF3 |  |  | 3000 | 0.022 | 0.1938 | No |
| 76 | PSMB2 |  |  | 3030 | 0.021 | 0.1919 | No |
| 77 | C1QBP |  |  | 3048 | 0.021 | 0.1914 | No |
| 78 | HNRNPD |  |  | 3359 | 0.014 | 0.1563 | No |
| 79 | ABCE1 |  |  | 3395 | 0.013 | 0.1532 | No |
| 80 | HNRNPR |  |  | 3452 | 0.012 | 0.1475 | No |
| 81 | ACP1 |  |  | 3454 | 0.012 | 0.1482 | No |
| 82 | AIMP2 |  |  | 3476 | 0.012 | 0.1466 | No |
| 83 | POLE3 |  |  | 3486 | 0.012 | 0.1463 | No |
| 84 | SMARCC1 |  |  | 3546 | 0.010 | 0.1402 | No |
| 85 | SNRPA |  |  | 3569 | 0.010 | 0.1383 | No |
| 86 | DUT |  |  | 3596 | 0.009 | 0.1360 | No |
| 87 | RNPS1 |  |  | 3725 | 0.006 | 0.1215 | No |
| 88 | TCP1 |  |  | 3757 | 0.006 | 0.1183 | No |
| 89 | FBL |  |  | 3777 | 0.005 | 0.1165 | No |
| 90 | NDUFAB1 |  |  | 3836 | 0.004 | 0.1100 | No |
| 91 | EIF3D |  |  | 3862 | 0.003 | 0.1074 | No |
| 92 | RAD23B |  |  | 3871 | 0.003 | 0.1066 | No |
| 93 | YWHAE |  |  | 3963 | 0.001 | 0.0962 | No |
| 94 | PPIA |  |  | 3964 | 0.001 | 0.0962 | No |
| 95 | PGK1 |  |  | 3973 | 0.001 | 0.0954 | No |
| 96 | CDC20 |  |  | 3996 | 0.001 | 0.0929 | No |
| 97 | CAD |  |  | 4107 | -0.001 | 0.0802 | No |
| 98 | EXOSC7 |  |  | 4207 | -0.003 | 0.0689 | No |
| 99 | SF3A1 |  |  | 4304 | -0.005 | 0.0581 | No |
| 100 | MRPS18B |  |  | 4306 | -0.005 | 0.0584 | No |
| 101 | CCT5 |  |  | 4370 | -0.006 | 0.0515 | No |
| 102 | MCM7 |  |  | 4435 | -0.008 | 0.0446 | No |
| 103 | SNRPD1 |  |  | 4477 | -0.008 | 0.0404 | No |
| 104 | NOP16 |  |  | 4691 | -0.012 | 0.0165 | No |
| 105 | EIF1AX |  |  | 4719 | -0.013 | 0.0143 | No |
| 106 | UBA2 |  |  | 4787 | -0.014 | 0.0075 | No |
| 107 | TUFM |  |  | 4837 | -0.015 | 0.0028 | No |
| 108 | GNL3 |  |  | 4977 | -0.018 | -0.0121 | No |
| 109 | PSMD3 |  |  | 4992 | -0.018 | -0.0125 | No |
| 110 | EIF4E |  |  | 5088 | -0.020 | -0.0221 | No |
| 111 | HDAC2 |  |  | 5329 | -0.025 | -0.0482 | No |
| 112 | NCBP2 |  |  | 5373 | -0.026 | -0.0514 | No |
| 113 | RRP9 |  |  | 5380 | -0.026 | -0.0503 | No |
| 114 | NPM1 |  |  | 5412 | -0.027 | -0.0521 | No |
| 115 | SNRPA1 |  |  | 5428 | -0.027 | -0.0519 | No |
| 116 | ORC2 |  |  | 5449 | -0.027 | -0.0523 | No |
| 117 | HNRNPA3 |  |  | 5463 | -0.028 | -0.0519 | No |
| 118 | TOMM70 |  |  | 5472 | -0.028 | -0.0509 | No |
| 119 | NME1 |  |  | 5479 | -0.028 | -0.0496 | No |
| 120 | CTPS1 |  |  | 5522 | -0.029 | -0.0525 | No |
| 121 | VDAC1 |  |  | 5528 | -0.029 | -0.0510 | No |
| 122 | SSB |  |  | 5596 | -0.030 | -0.0567 | No |
| 123 | PSMD7 |  |  | 5599 | -0.031 | -0.0548 | No |
| 124 | RUVBL2 |  |  | 5610 | -0.031 | -0.0538 | No |
| 125 | PRDX3 |  |  | 5634 | -0.031 | -0.0543 | No |
| 126 | SRM |  |  | 5656 | -0.032 | -0.0545 | No |
| 127 | HPRT1 |  |  | 5723 | -0.033 | -0.0598 | No |
| 128 | BUB3 |  |  | 5807 | -0.034 | -0.0671 | No |
| 129 | EIF2S1 |  |  | 5897 | -0.036 | -0.0749 | No |
| 130 | TRIM28 |  |  | 5969 | -0.037 | -0.0805 | No |
| 131 | CSTF2 |  |  | 6005 | -0.038 | -0.0819 | No |
| 132 | HDDC2 |  |  | 6205 | -0.043 | -0.1021 | No |
| 133 | SRSF2 |  |  | 6250 | -0.044 | -0.1041 | No |
| 134 | TRA2B |  |  | 6303 | -0.045 | -0.1071 | No |
| 135 | RANBP1 |  |  | 6309 | -0.045 | -0.1045 | No |
| 136 | CDC45 |  |  | 6326 | -0.045 | -0.1032 | No |
| 137 | CYC1 |  |  | 6383 | -0.047 | -0.1065 | No |
| 138 | PSMC6 |  |  | 6525 | -0.050 | -0.1193 | No |
| 139 | XRCC6 |  |  | 6553 | -0.052 | -0.1188 | No |
| 140 | TFDP1 |  |  | 6587 | -0.052 | -0.1190 | No |
| 141 | SRSF7 |  |  | 6593 | -0.053 | -0.1159 | No |
| 142 | SYNCRIP |  |  | 6600 | -0.053 | -0.1129 | No |
| 143 | PTGES3 |  |  | 6602 | -0.053 | -0.1093 | No |
| 144 | HSP90AB1 |  |  | 6619 | -0.053 | -0.1074 | No |
| 145 | VBP1 |  |  | 6654 | -0.054 | -0.1076 | No |
| 146 | SRSF1 |  |  | 6655 | -0.054 | -0.1038 | No |
| 147 | ETF1 |  |  | 6699 | -0.056 | -0.1049 | No |
| 148 | MCM5 |  |  | 6765 | -0.058 | -0.1084 | No |
| 149 | SRPK1 |  |  | 6891 | -0.062 | -0.1186 | No |
| 150 | EIF3B |  |  | 6912 | -0.062 | -0.1166 | No |
| 151 | HNRNPU |  |  | 7074 | -0.067 | -0.1306 | No |
| 152 | G3BP1 |  |  | 7109 | -0.068 | -0.1298 | No |
| 153 | VDAC3 |  |  | 7150 | -0.069 | -0.1296 | No |
| 154 | COX5A |  |  | 7157 | -0.070 | -0.1254 | No |
| 155 | IFRD1 |  |  | 7168 | -0.070 | -0.1216 | No |
| 156 | APEX1 |  |  | 7171 | -0.070 | -0.1170 | No |
| 157 | PA2G4 |  |  | 7192 | -0.071 | -0.1143 | No |
| 158 | RAN |  |  | 7221 | -0.072 | -0.1126 | No |
| 159 | SERBP1 |  |  | 7240 | -0.072 | -0.1096 | No |
| 160 | PSMC4 |  |  | 7254 | -0.073 | -0.1060 | No |
| 161 | UBE2L3 |  |  | 7292 | -0.074 | -0.1051 | No |
| 162 | RSL1D1 |  |  | 7349 | -0.076 | -0.1063 | No |
| 163 | SNRPD3 |  |  | 7385 | -0.077 | -0.1050 | No |
| 164 | HSPD1 |  |  | 7433 | -0.079 | -0.1049 | No |
| 165 | KPNB1 |  |  | 7453 | -0.080 | -0.1015 | No |
| 166 | EIF4A1 |  |  | 7459 | -0.080 | -0.0965 | No |
| 167 | RRM1 |  |  | 7491 | -0.081 | -0.0945 | No |
| 168 | DHX15 |  |  | 7536 | -0.083 | -0.0938 | No |
| 169 | PSMA1 |  |  | 7678 | -0.090 | -0.1039 | No |
| 170 | PSMD8 |  |  | 7746 | -0.093 | -0.1052 | No |
| 171 | SNRPB2 |  |  | 7755 | -0.093 | -0.0996 | No |
| 172 | MRPL9 |  |  | 7816 | -0.097 | -0.0998 | No |
| 173 | MCM4 |  |  | 7900 | -0.101 | -0.1023 | No |
| 174 | SSBP1 |  |  | 7928 | -0.103 | -0.0983 | No |
| 175 | HNRNPA2B1 |  |  | 7941 | -0.103 | -0.0924 | No |
| 176 | PSMA4 |  |  | 7995 | -0.107 | -0.0911 | No |
| 177 | PRPF31 |  |  | 8015 | -0.108 | -0.0858 | No |
| 178 | CDK2 |  |  | 8018 | -0.108 | -0.0784 | No |
| 179 | KPNA2 |  |  | 8045 | -0.110 | -0.0737 | No |
| 180 | PCNA |  |  | 8061 | -0.111 | -0.0677 | No |
| 181 | MCM2 |  |  | 8099 | -0.114 | -0.0641 | No |
| 182 | PSMA2 |  |  | 8122 | -0.116 | -0.0585 | No |
| 183 | DDX21 |  |  | 8172 | -0.119 | -0.0559 | No |
| 184 | XPO1 |  |  | 8216 | -0.123 | -0.0523 | No |
| 185 | CCNA2 |  |  | 8227 | -0.124 | -0.0448 | No |
| 186 | LDHA |  |  | 8261 | -0.127 | -0.0397 | No |
| 187 | TYMS |  |  | 8287 | -0.131 | -0.0335 | No |
| 188 | PSMD1 |  |  | 8396 | -0.146 | -0.0358 | No |
| 189 | PSMB3 |  |  | 8416 | -0.148 | -0.0276 | No |
| 190 | MAD2L1 |  |  | 8427 | -0.150 | -0.0183 | No |
| 191 | ILF2 |  |  | 8579 | -0.181 | -0.0232 | No |
| 192 | NOP56 |  |  | 8619 | -0.199 | -0.0138 | No |
| 193 | PRDX4 |  |  | 8660 | -0.217 | -0.0032 | No |
| 194 | CANX |  |  | 8735 | -0.270 | 0.0071 | No |
Table: GSEA details [plain text format]

  

Fig 2: HALLMARK\_MYC\_TARGETS\_V1: Random ES distribution      
 Gene set null distribution of ES for **HALLMARK\_MYC\_TARGETS\_V1**
